# Quantitative Understanding of Nanoparticle Uptake in Watermelon Plants

**DOI:** 10.3389/fpls.2016.01288

**Published:** 2016-08-26

**Authors:** Ramesh Raliya, Christina Franke, Sanmathi Chavalmane, Remya Nair, Nathan Reed, Pratim Biswas

**Affiliations:** ^1^Department of Energy, Environmental and Chemical Engineering, Washington University in St. LouisSt. Louis, MO, USA; ^2^Department of Biomedical Engineering, Case Western Reserve UniversityCleveland, OH, USA

**Keywords:** aerosol delivery, biointerface, gold nanoparticle, internalization, nanoparticle, watermelon

## Abstract

The use of agrochemical-nutrient fertilizers has come under scrutiny in recent years due to concerns that they damage the ecosystem and endanger public health. Nanotechnology offers many possible interventions to mitigate these risks by use of nanofertilizers, nanopesticides, and nanosensors; and concurrently increases profitability, yields, and sustainability within the agricultural industry. Aerosol based foliar delivery of nanoparticles may help to enhance nanoparticle uptake and reduce environmental impacts of chemical fertilizers conventionally applied through a soil route. The purpose of this work was to study uptake, translocation, and accumulation of various gold nanostructures, 30–80 nm, delivered by aerosol application to a watermelon plant. Cellular uptake and accumulation of gold nanoparticles were quantified by Inductively Coupled Plasma-Mass Spectroscopy (ICP-MS). Observations suggested that nanoparticles could be taken up by the plant through direct penetration and transport through the stomatal opening. Observed translocation of nanoparticles from leaf to root shows evidence that nanoparticles travel by the phloem transport mechanism. Accumulation and transport of nanoparticles depend on nanoparticle shape, application method, and nature of plant tissues.

## Introduction

The application of nanotechnology in the area of plant sciences has been extensively studied in recent years (Peng et al., 2012; Chen et al., 2014). A key aspect of the work of plant nutritional scientists is toward the precise delivery of nutrients and enhanced nutrient uptake. Chemical fertilizer uptake efficiency in plants is low due to fixation of nutrients with other soil composites or run off due to precipitation leading to a growing anthropogenic eutrophication issue (Smith et al., 1999; Hautier et al., 2014). To address these challenges, nanoparticles have been researched for use as insecticides (Wibowo et al., 2014), fungicides (Becker et al., 2010; Capaldi Arruda et al., 2015; Saharan et al., 2015) and nanofertilizers (DeRosa et al., 2010; Raliya and Tarafdar, 2013; Liu and Lal, 2014, 2015; Raliya et al., 2014, 2016; Tarafdar et al., 2014). While there has been a significant push to engineer beneficial nanoparticles, there are also unintentional exposures and releases of nanoparticles from various industries that may end up in the ecosystem (Biswas and Wu, 2005; Yang et al., 2014). It is well reported that internalization and subsequent toxicity of nanoparticles within *in vitro* microenvironments depend on chemical composition, particle size, and surface chemistry that affects ligand-receptor interactions. For instance, Jiang et al. (2008) established size dependent reactive oxygen species (ROS) generation caused by TiO_2_ nanoparticles. The highest ROS activity per unit area was observed for 30 nm particles, and observed to be constant above 30 nm. Similarly, Albanese et al. (2012) provide a rationale by correlating the properties of nanomaterials such as size, shape, chemical functionality, surface charge, and composition with biomolecular signaling, biological kinetics, transport and toxicity in cell culture studies. However, there is no study that correlates morphology dependent nanoparticle uptake to an application method.

Nair et al. (2010) and González-Melendi et al. (2008) reviewed the delivery of nanoparticulate materials to plants and provided evidence of internalization by various microscopic techniques. Unfortunately, most microscopic imaging only allows for a qualitative investigation of a minute fraction of plant tissues ranging between nanometer to micrometer size scale. Further biasing can occur during staining of plant intracellular bodies for electron microscopy due to nanomaterial losses. Therefore, the tiny fraction of plant tissue examined may not necessarily represent the whole plant. Consequently, interactions of nanoparticles with plants are possible in many ways, and their impact on crop plant and food safety need to be investigated systemically.

Inductively coupled plasma—mass spectroscopy (ICP-MS) or inductively coupled plasma—optical emission spectroscopy (ICP-OES) are powerful tools to analyze metal accumulation in plants. In both the techniques, the entire plant tissue digested in the acid mixture before analysis. ICP-MS is preferred for nanoparticle detection in biological samples, due its detection sensitivity (Scheffer et al., 2008). During ICP-MS analysis, digested samples are introduced into argon plasma as aerosol droplets. The droplets dry in the plasma region, dissociate into the molecular constituents, and then form singly-charged ions, which are directed to a filtering device known as the mass spectrometer.

In the present study, gold nanoparticles (Au NPs) were used due to their biologically inert properties and their past utilization in genetic engineering through adsorbtion of DNA on a gold particle surface which can be delivered to cells (Ghosh et al., 2008; Thakor et al., 2011). Au NPs have shown great promise in the facile, on-site detection of contagious plant viruses when applied to a strip sensor (Zhao et al., 2011; Wei et al., 2014). These nanoparticles can be functionalized to target specific sites within an organism's cellular substructure, and could allow genetic modifications to be made from within the plant itself, rather than through a traditional *in-vitro* process. Moreover, if dispersed throughout a plant, Au NPs may eventually prove useful for real-time plant disease detection. Nonetheless, it is essential to understand the fundamental mechanisms of morphology-dependent cellular uptake of gold nanoparticles, as well as their transport and subsequent fate in a plant system.

In this work, we describe the effects of gold nanostructure on their internalization, translocation and accumulation in the watermelon plant. Au NPs of various sizes and morphologies were synthesized, characterized, and applied to the plants in aqueous suspension via foliar (drop-cast and aerosol) application. The objectives of this study were (1) to optimize nanoparticle delivery in plants comparing conventional drop cast approach v/s aerosol technique (2) to understand the uptake, transport and accumulation of nanoparticles in correlation to nanoparticle morphology and (3) to develop an alternative approach of nanoparticle quantification in plant tissue using ICP-MS. Au NPs were chosen as a representative of metallic nanoparticles because of its broad application and ease to tune particle morphology. Watermelon plant (*Citrullus lanatus*) was chosen because of its popularity as an edible fruit around the world, and also because it has leaves with both large stomata and vessel size, which may facilitate nanoparticle uptake and translocation.

## Materials and methods

The experimental plan of the study is summarized in Table 1, and details of the same are described in the following sections.

**Table 1:** **Test plan for the overall experiment**.

**No**.	**Experiment**	**Objective**	**Investigation Finding**	**Notes**
1	Synthesis of Au nanostructures	To produce nanostructures with varying morphologies	Sphere, cube, rhombic decahedral, and rod shape Au nanostructures were synthesized in the range of 30–90 nm	Morphology of Au nanostructures were confirmed by UV-VIS spectroscopy, TEM
2	Application of synthesized nanostructures to plants	To study Au nanostructure–watermelon plant interaction	Aqueous suspensions of AuNPs were diluted to 100 ppm and applied to watermelon leaves	Two foliar application methods were used: aerosol and drop-cast methods
3	Elemental analysis of Au concentrations in plant	To compare the efficacy of uptake, translocation and accumulation of various Au nanostructures	Spherical particles translocated to greatest extent by aerosol method; Rods translocated to greatest extent by drop-cast method	Elemental analysis performed using ICP-MS

### Gold nanoparticle synthesis and characterization

#### Reagents

Ascorbic acid (99.8%) was procured from J.T. Baker. Hexadecyltrimethyl ammonium chloride (>95.0%) was purchased from Tokyo Chemical Industry Co, Ltd. odium borohydride (≥99%), hexadecyltrimethyl ammonium bromide (≥99.0%), sodium bromide (≥99.0%), silver nitrate (99.9999%), gold (III) chloride trihydrate (≥99.9%), and gold chloride solution (200 mg/dL) were purchased from Sigma-Aldrich. All chemicals were used as received.

#### Synthesis

Gold nanoparticles of spherical, cubic, rhombic dodecahedral (RD), and rod morphologies were prepared using seed-mediated methods (Becker et al., 2010; Wu et al., 2010). Gold seeds (2–3 nm) were produced through the reaction of gold chloride with the reductant, ascorbic acid. Formation of gold seed particles was evident due to the immediate brown color formation upon addition of catalyst, sodium borohydride. Au NPs formation occurs as a result of the nucleation of gold seeds in growth solutions, varying the amount of gold seeds and concentration of reducing agent.

#### Characterization

Synthesized nanoparticles were characterized for their morphological properties prior to their application to the watermelon plants.

#### Transmission electron microscopy (TEM)

Nanoparticle samples were prepared for TEM characterization by placing a drop of aqueous particle suspension on a carbon-coated copper grid and allowing the sample to air-dry. TEM images were viewed and obtained with the aid of a Tecnai G^2^ Spirit transmission electron microscope (FEI, USA).

#### UV-visible (UV-Vis) spectrophotometry

Nanoparticle samples were diluted 2X with deionized (DI) water for UV-Visible spectrophotometry analyses. Surface plasmon resonance of each sample was determined using a Varian Cary 50 UV-visible spectrophotometer (Varian, Inc., USA), measuring the absorbance of light with wavelengths in the 400–1000 nm range at medium sample speed. The maximum peak in each absorbance band was taken to be the surface plasmon resonance of the sample.

#### Dynamic light scattering (DLS)

Samples were prepared for DLS characterization by diluting 2X with DI water. Using DLS, hydrodynamic size and zeta potential measurements were performed using a Malvern Zeta Sizer Nano ZS (Malvern Instruments, USA). Samples were equilibrated for 2 min at 25°C before obtaining each measurement. All the measurements were performed in triplicate.

#### Inductively-coupled mass spectrometry (ICP-MS)

To determine the nanoparticle concentration in suspension, 100 μL of each nanoparticle solution was digested in 400 μL aqua regia (Nitric acid: hydrochloric acid, 3:1). Digested samples were diluted with 4.5 mL 1% nitric acid. Elemental concentration of gold in each sample was measured with the aid of an ELAN DRC II ICP-MS (Perkin Elmer, Inc., USA). The concentrations of spheres, cubes, RD, and rods (in ppm) were 2.29 × 10^3^, 1.12 × 10^4^, 1.18 × 10^4^, and 1.44 × 10^3^, respectively (Table 2).

**Table 2:** **Characterization data for nanoparticles in aqueous suspension**.

**Particle Morphology**	**Particle Size^a^ (nm)**	**Hydrodynamic Size^c^ (nm)**	**PDI^c^, ^d^**	***λ_max_*^e^ (nm)**	**Zeta Potential^c^ (mV)**	**Concentration, Au (ppm)**
Sphere	35 ± 4.2^*^	61.0 ± 1.6	0.457 ± 0.02	527	−40.5 ± 2.3	2.29 × 103
Truncated Cube	70 ± 2.6	99.2 ± 25.1	0.034 ± 0.001	563	56.7 ± 6.6	1.12 × 104
Rhombic Dodecahedra	65 ± 3.4	99.8 ± 30.4	0.066 ± 0.01	550	64.3 ± 4.2	1.18 × 104
Rod	20 ± 0.92, 60 ± 1.3^b^	114.1 ± 52.1	0.296 ±0.03	816	43.9 ± 3.3	1.44 × 103
Replicate (*n*)	*n* = 50	*n* = 5	*n* = 5	−	*n* = 5	−

a Mean geometric diameter, obtained from TEM images.

b Diameter, length.

c From DLS, number based particle size distribution.

d Polydispersity index.

e Surface Plasmon Resonance, obtained from maximum UV-vis peak.

* Standard error from the mean value.

#### Watermelon plants and growth conditions

All plants were grown in a controlled environment chamber with a constant temperature of 34 ± 2°C, 60 ± 2% relative humidity, constant air flow, a photoperiod of 16:8 h and a photosynthetic photon flux density of 750 μmol m^−2^ s^−1^. Black Diamond watermelon (*C. lanatus*) seeds were grown in plastic pots (38 mm square) filled with moisture control potting mix (Miracle-Gro Lawn Products, USA). One seed was planted in each pot. Soil was kept moist and pots were covered until seed germination. Upon germination, pot covers were removed and seedlings were given 10 ml deionized water daily. In addition, 5 mL of nutrient solution mixture (Miracle Grow, USA) was also supplied on every alternate day.

#### Exposure of nanoparticles to plant

Each nanoparticle sample was diluted with DI water to 100 ppm for spraying. To prepare watermelon plants for spraying, all leaves were left intact, but one was removed to function as a control. Experiments were performed in triplicate for each type of particle and each application method, and four plants, each of 14 days old were used for control, on which only DI water was applied. All the exposure experiments were conducted in between 11 a.m. and 1 p.m. to ensure stomatal opening.

#### Aerosol method

**Figure 1:**
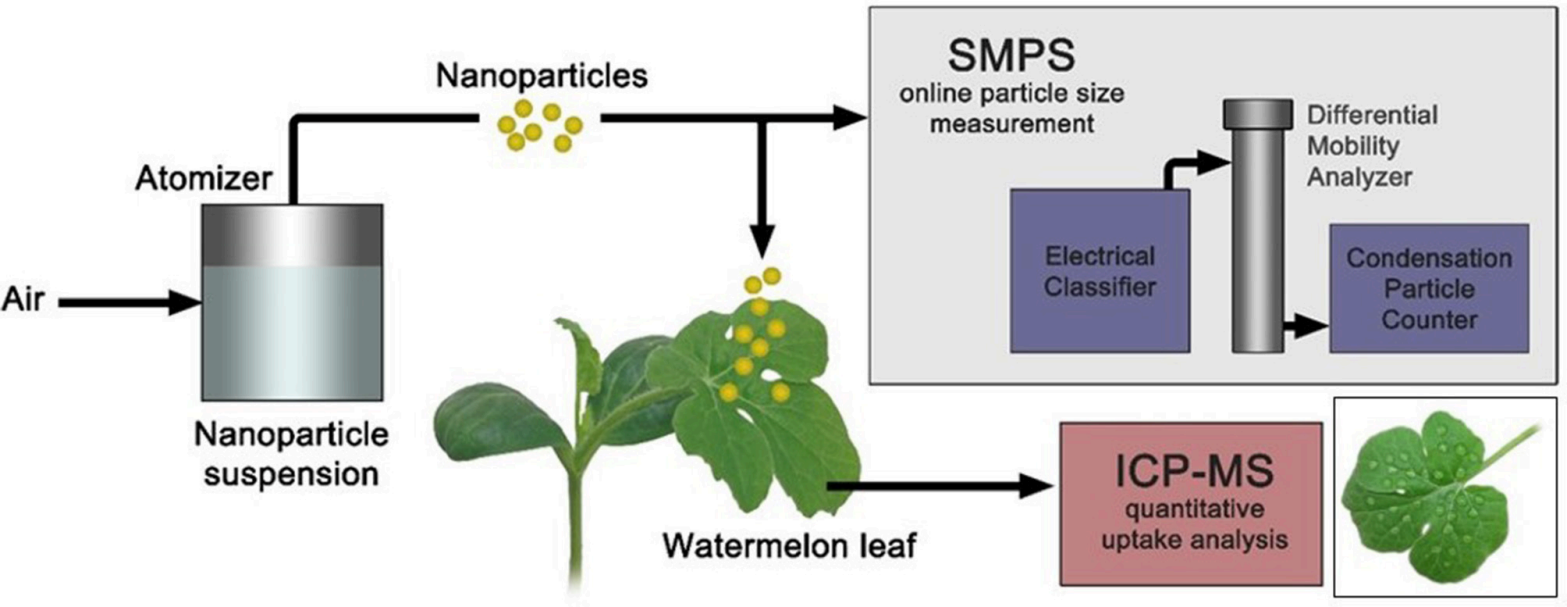
**Schematic of an aerosol method for nanoparticle delivery and quantification of nanoparticle uptake, accumulation, and translocation in watermelon**. Bottom right image represents drop caste method of nanoparticle delivery to leaf using auto pipette.

A schematic of the atomizer application method is provided in Figure 1. The soil of the plants was covered so that no particles could be sprayed directly onto the soil. The surface of each plant's remaining mature leaf was sprayed for 10 min in a fume hood. Gold nanoparticles were aerosolized by a TSI atomizer under 30 psi flowing air.

During exposure, online measurements of the applied particle size and number concentration were monitored by Scanning Mobility Particle Sizer (SMPS, TSI, Inc., USA). Aerosol distributions are predominantly lognormal in character, so data is typically plotted on a lognormal X-axis. In the simplest technique, particle data is plotted as a function of the concentration (dN) for each particle size bin. The mode concentration of the size distribution is estimated by the concentration in the peak bin. dN (or ΔN) is the number of particles in the range (total concentration) and dlogDp (or ΔlogDp) is the difference in the log of the channel width. dlogDp is calculated by subtracting the log of the lower bin boundary from the log of the upper boundary for each channel (normalizing for bin width). The concentration is divided by the bin width, giving a normalized concentration value that is independent of the bin width (TSI Inc., USA) using following formula by the TSI software.

$$\text{dN}/d\text{​}\log\text{​}Dp=\frac{dN}{d\text{​}\log\text{​}Dp}=\frac{dN}{\log\text{​}Dp,\;u-\log\text{​}Dp,l}$$

Where,

$$\begin{array}{lll} \text{dN} & = & \text{particle concentration}\\ \text{Dp} & = & \text{midpoint particle diameter}\\ \text{Dp,u} & = & \text{upper channel diameter}\\ \text{Dp,l} & = & \text{Lower channel diameter} \end{array}$$

#### Drop-cast method

In order to compare the nanoparticle uptake with respect to aerosol application, equal amount of each sample was placed in small droplets using auto pipette on the surface of a plant's mature leaf. Plants were left undisturbed after applying particles, allowing the droplets to air dry. An image of a leaf after treatment by the drop-cast method is provided in Figure 1.

#### Nanoparticle uptake, transport, and accumulation analysis using ICP-MS

After nanoparticle exposure to plants either by an aerosol or drop cast method, plants were allowed to grow for 48 h in the environmental condition described above. This allowed accumulation of the nanoparticles as they were transported and interacted with plant cells and tissues.

#### Harvest plants

Plants were harvested 48 h after applying the nanoparticles. To prepare plant samples for uptake analysis, roots were first washed with tap water to remove the adhered soil. The entire plant was then rinsed with DI water three times. Roots, stems, seed leaves, and sprayed leaves of each plant were separated into 20 ml glass vials. The harvested samples were placed in a drying oven at 60°C until the dry matter reached a constant weight.

#### Elemental analysis

After all samples had dried for 60–72 h at 60°C, each sample was crushed into a fine powder. Powdered samples (100 mg) were digested in 6 mL aqua regia (HNO_3_ and HCl) at 150°C using microwave digestion (CEM MARS 6 Xpress, CEM Corp., USA). After complete digestion, each sample was suspended in 5 mL DI water and filtered through a 25 mm syringe filter with a 0.45 μm nylon membrane (VWR Inc., USA). These filtered samples were analyzed with the aid of an ELAN DRC II ICP-MS (Perkin Elmer, Inc., USA) to determine the concentration of elemental gold in each plant section. Based on the raw data of elemental detection intensity, nanoparticles uptake and accumulation were calculated by the ELAN DRC II ICP MS software.

#### Statistical analyses

The results were expressed as mean ± SD (standard deviation); *n* = 5 (except nanoparticle size data given in Table 2, where *n* was 50). Statistical analyses was performed using data analyses function of Microsoft Excel V.2013. The significant difference in the same concentration of gold nanoparticle exposure (for individual shape) applied by drop-cast or aerosol method were analyzed by Student *t*-test. A *p*-value of less than 0.05 (*p* < 0.05) was considered as statistically significant.

## Results and discussion

**Figure 2:**
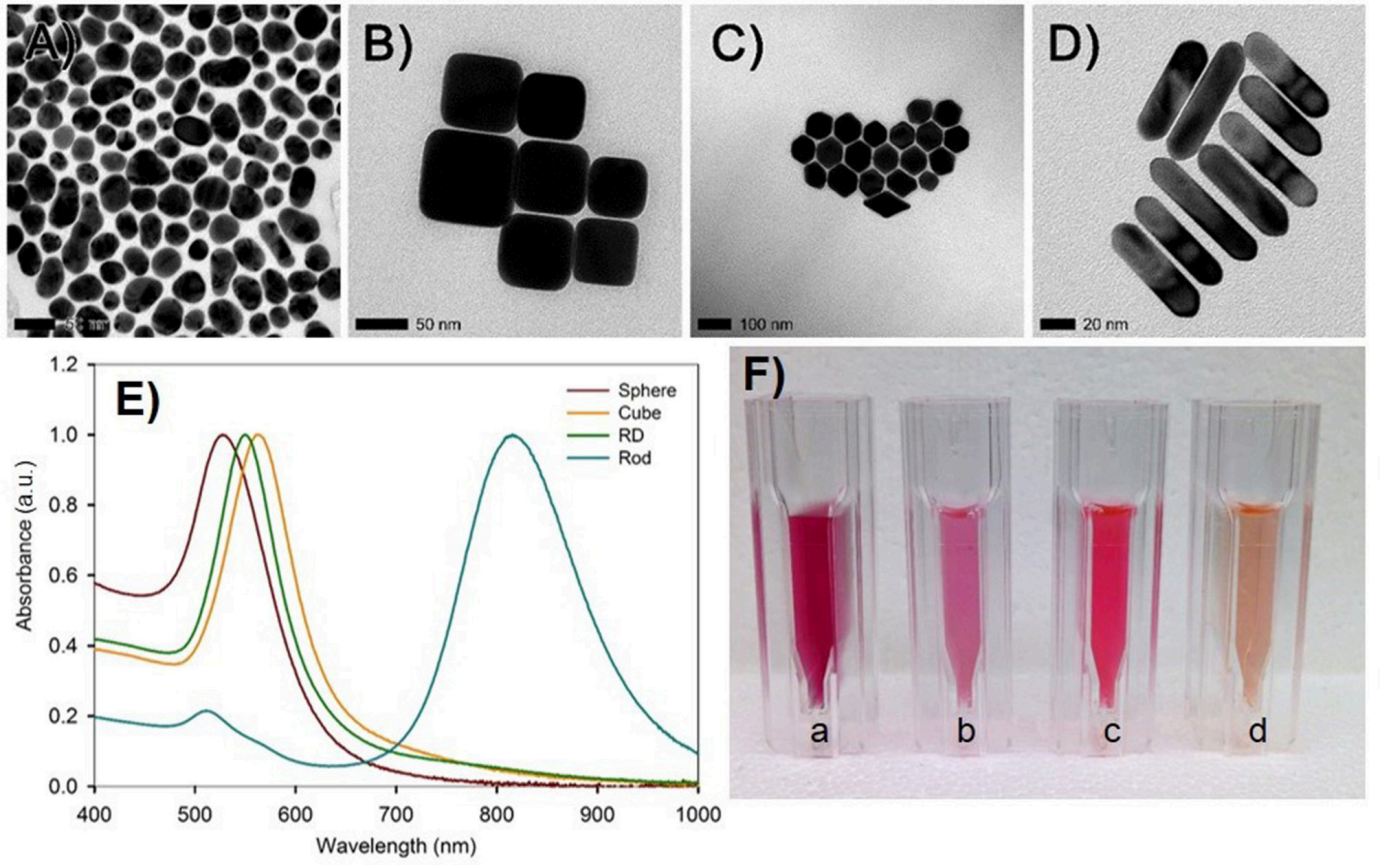
**Characterization of gold nanoparticles**. TEM images of gold nanostructure **(A)** spheres, **(B)** truncated cubes, **(C)** rhombic dodecahedra, and **(D)** rods. **(E)** UV-visible absorption spectra showing characteristic absorption for each nanostructure. **(F)** Pictured from left (a) to right (d): nano-spheres, truncated cubes, rhombic dodecahedra, and rods as synthesized in aqueous suspension.

The particle characterization results are presented first, followed by a description of their impact on the plants. As indicated in test plan 1 (Table 1), particle morphologies, surface chemistry, and geometric diameters were determined to study their uptake and transport by watermelon (Figures 2A–D). Mean geometric diameters of spherical, cubic, and RD particles were found to be 35, 70, and 65 nm respectively (Table 2). Rod-shaped particles were 60 nm in length, 20 nm in diameter, and therefore with an aspect ratio of three (Table 2). The morphology and diameter of Au NPs were also evidenced by UV-Vis absorption spectra and specific color intensities of the solutions (Figures 2E,F).

**Figure 3:**
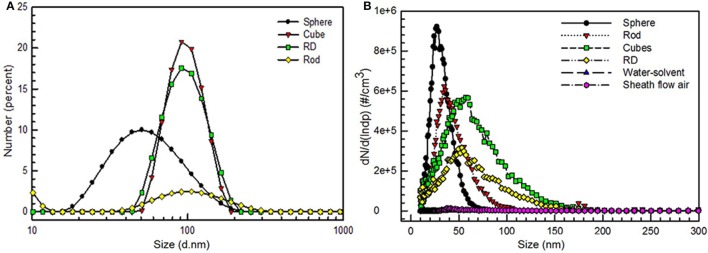
**Online and offline size measurement of gold nanostructures**. **(A)** Hydrodynamic size measurements performed using light scattering (DLS) technique. **(B)** Online measurements of number concentrations of particles, monitored by Scanning Mobility Particle Sizer (SMPS).

Hydrodynamic size and zeta potential values were measured by the offline tool, DLS, and are summarized in Table 2. Zeta potential of the nanoparticles were determined to be −40.5, 56.7, 64.3, and 43.9 mV for spheres, cubes, RD, and rods respectively. The hydrodynamic sizes of all particles were larger than the geometric mean diameters determined from TEM images. This size disparity is due to the soft agglomeration of nanoparticles due to Van der Waals forces (Wang et al., 2013). Therefore, by comparing these two foliar application methods, we optimized the effect of applied droplet size for maximum Au NPs uptake in plants. In order to precisely determine nanoparticle delivery, the size distribution of the gold nanoparticles were monitored in real time using a (SMPS). Before each exposure, a background measurement of particle size distributions of air and DI water was carried out. The results (base lines in the Figure 3B) show that DI water and air do not have any significant particle concentration or formation, thus implying a negligible effect on the aerosol size and number concentration measured by the SMPS. The SMPS measurements showed good agreement with the geometric mean diameter (GMD) observed by TEM (Figures 3A,B). The observations indicate that shear forces in the atomizer during aerosolization resulted in the break-up of Au NP agglomerates and the formation of singlet particles (Brandt et al., 1987).

The nanoparticle suspensions were aerosolized and delivered to the watermelon leaf surface. For comparison, a solution drop method was employed concurrently for all the sample material in equal exposure concentration and volume. After 48 h all the samples were harvested and analyzed by ICP-MS to detect Au NPs concentration in leaves, stem and roots. The measured concentrations of elemental gold in each sample were normalized by the dried mass of the plant section, and this data was processed to determine the percent of recovered gold in each plant section.

**Figure 4:**
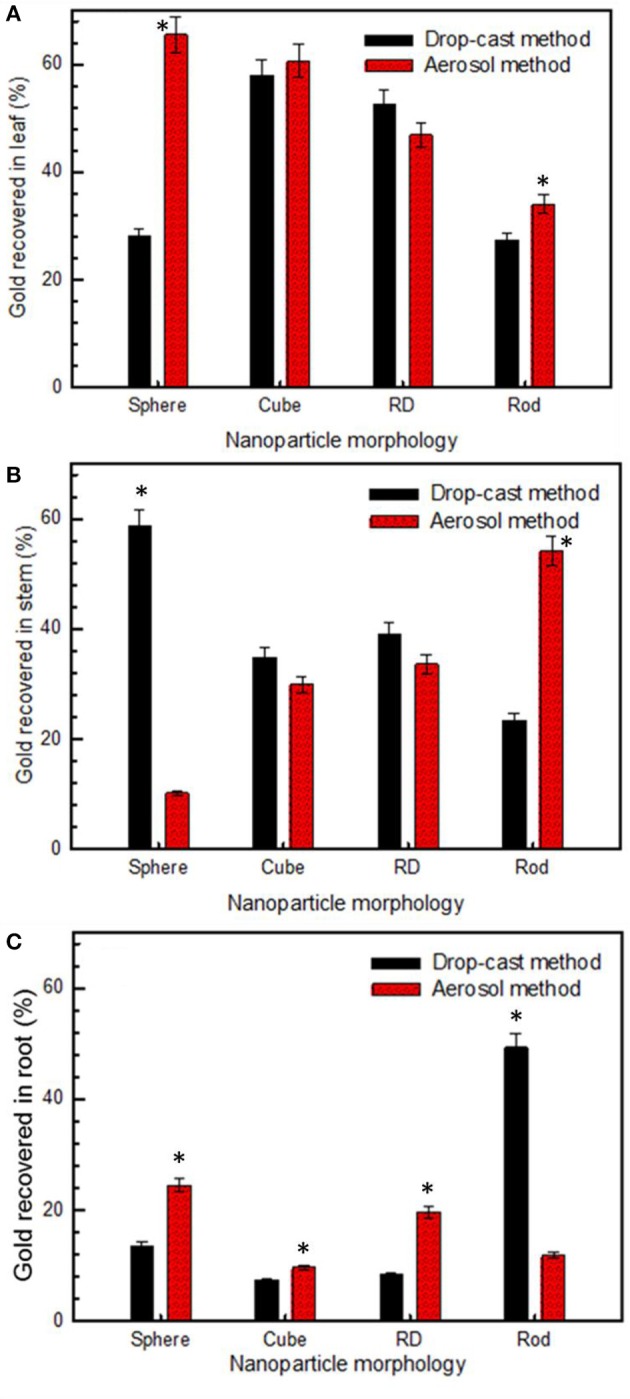
**Gold nanoparticles accumulation in watermelon that was recovered by ICP-MS in (A) leaf, (B) stem, and (C) root sections of treated plants, comparing results of drop-cast and aerosol method of nanoparticle delivery (*n* = 4)**. Asterisk(s) above bar demonstrate significant difference (*p* < 0.05).

ICP-MS analyses suggest that, when applied by drop-cast method, 28.0% of spherical particles were accumulated in the leaf, followed by 58.6% recovered in the stem and 13.4% in the root (Table 3). Comparing the results of the two application methods, aerosol application of low aspect ratio particles (sphere, cube, and rhombic dodecahedra) enhanced their transport, resulting in a greater percentage of gold being recovered in the root sections. For sphere, cube, and RD morphologies, the increase in translocation rate of the aerosolized vs. drop-cast nanoparticles was 10.9, 2.3, and 11.3% respectively, whereas a 37.4% decrease in translocation rate was observed for the nanorods (Table 3). This trend suggests that the smaller droplet size delivered by the aerosol method increased translocation of low aspect ratio particles to the plant roots, but inhibited the translocation of higher aspect ratio nanoparticles. The opposite trend was observed for the larger droplet size delivered by the drop-cast method. When the nanoparticles were applied using the drop cast method, the rods, which had a higher aspect ratio than other morphologies tested, translocated to the roots to the greatest extent (49.2%) as compared to the translocation of spheres (13.4%), cubes (7.3%), and rhombic dodecahedra (8.3%; Figures 4A–C). Thus, the larger droplet size may improve the uptake and transport of high aspect ratio nanoparticles.

**Table 3:** **Elemental analysis results of gold in plant sections by ICP-MS**.

**Particle Morphology**	**Method**	**Amount in leaf (%)**	**Amount in stem (%)**	**Amount in root (%)**	Mean percent difference (drop-cast vs aerosol) in root^*^
Sphere	Drop-cast	28.0 ± 1.3^a^	58.6 ± 2.7	13.4 ± 0.3	10.9
	Aerosol	65.5 ± 1.7	10.1 ± 0.1	24.4 ± 1.1	
Cube	Drop-cast	57.9 ± 2.6	34.8 ± 1.7	7.3 ± 0.5	2.3
	Aerosol	60.6 ± 0.8	29.8 ± 0.9	9.6 ± 0.1	
Rhombic dodecahedra	Drop-cast	52.6 ± 1.4	39.1 ± 1.6	8.3 ± 0.1	11.3
	Aerosol	46.8 ± 1.8	33.6 ± 1.4	19.6 ± 0.5	
Rod	Drop-cast	27.4 ± 1.2	23.4 ± 0.8	49.3 ± 1.2	37.4
	Aerosol	34.0 ± 2.1	54.1 ± 1.7	11.8 ± 0.1	

* Standard error from the mean value.

* Translocation from leaf to root. The % difference is calculated by subtracting % amount of gold present in the root of drop cast delivered plant from the aerosol sprayed plant root.

**Figure 5:**
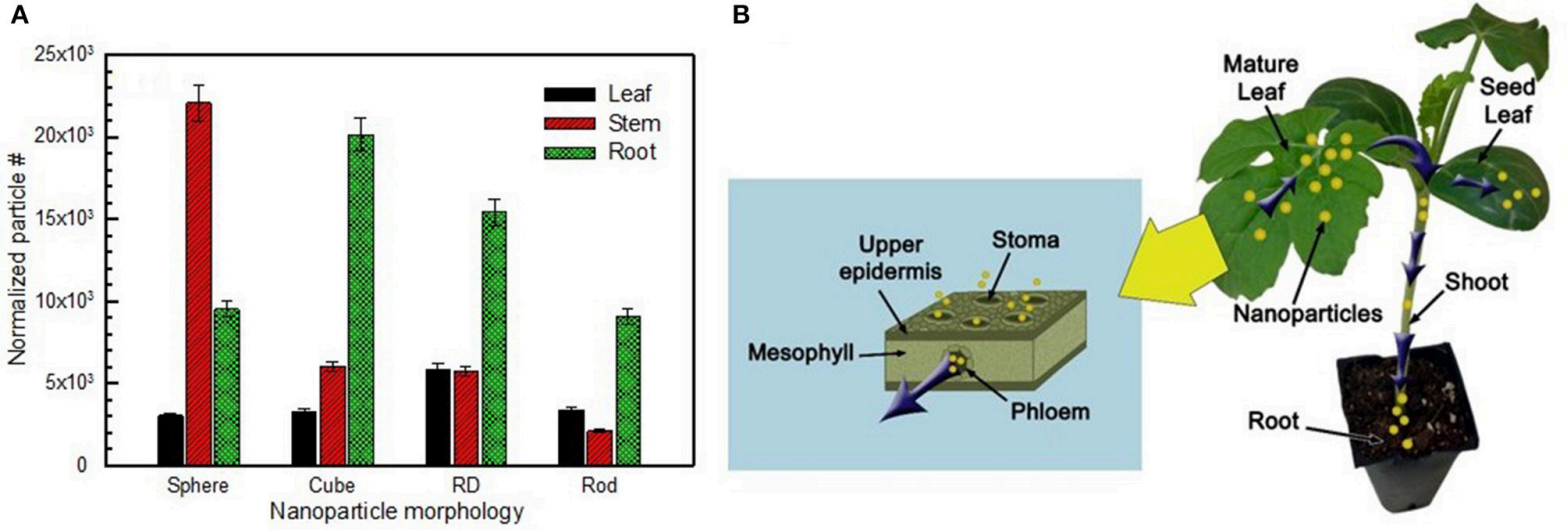
**Transport and accumulation of gold nanoparticles**. **(A)** Number of particles recovered in sections of aerosol-treated plants, normalized to SMPS number concentration of particles. **(B)** Schematic of translocation of nanoparticles from leaf to root by phloem transport. Inset: gas phase uptake of nanoparticles through stomata opening.

Furthermore, trends comparing all nanoparticles were identified. For drop-cast application methods, a trend in the efficacy of translocation (percent of recovered gold accumulated in roots) was observed as: rod (49.3%) > sphere (13.4%) > RD (8.3%) > cube (7.3%), whereas, trend for aerosol route applied particles were cube (37.38%) > RD (28.03%) > sphere (17.76%) and rod (16.82%). The results of the aerosol application method were further processed, normalizing the acquired concentration of gold by the online measurement of number concentration of particles in order to account for any discrepancies in the number of particles applied. From these normalized results (Figure 5A), a trend in the efficacy of translocation (number of particles recovered in root) was observed as: cube (20,000) > RD (15,000) > sphere (9500) > rod (9000), a trend which again suggests that the aerosol application method results in improved translocation of low aspect ratio particles. In this experiment, we did not see any recognizable stress responses in the treated plants, supporting our hypothesis that AuNP are nontoxic to plants due to their biologically inert state (Shukla et al., 2005). Notwithstanding, our procedure required only 48 h of AuNP exposure; therefore, to further verify this hypothesis, extended toxicological studies must be conducted to determine the long-term effects of Au NPs on plants.

**Figure 6:**
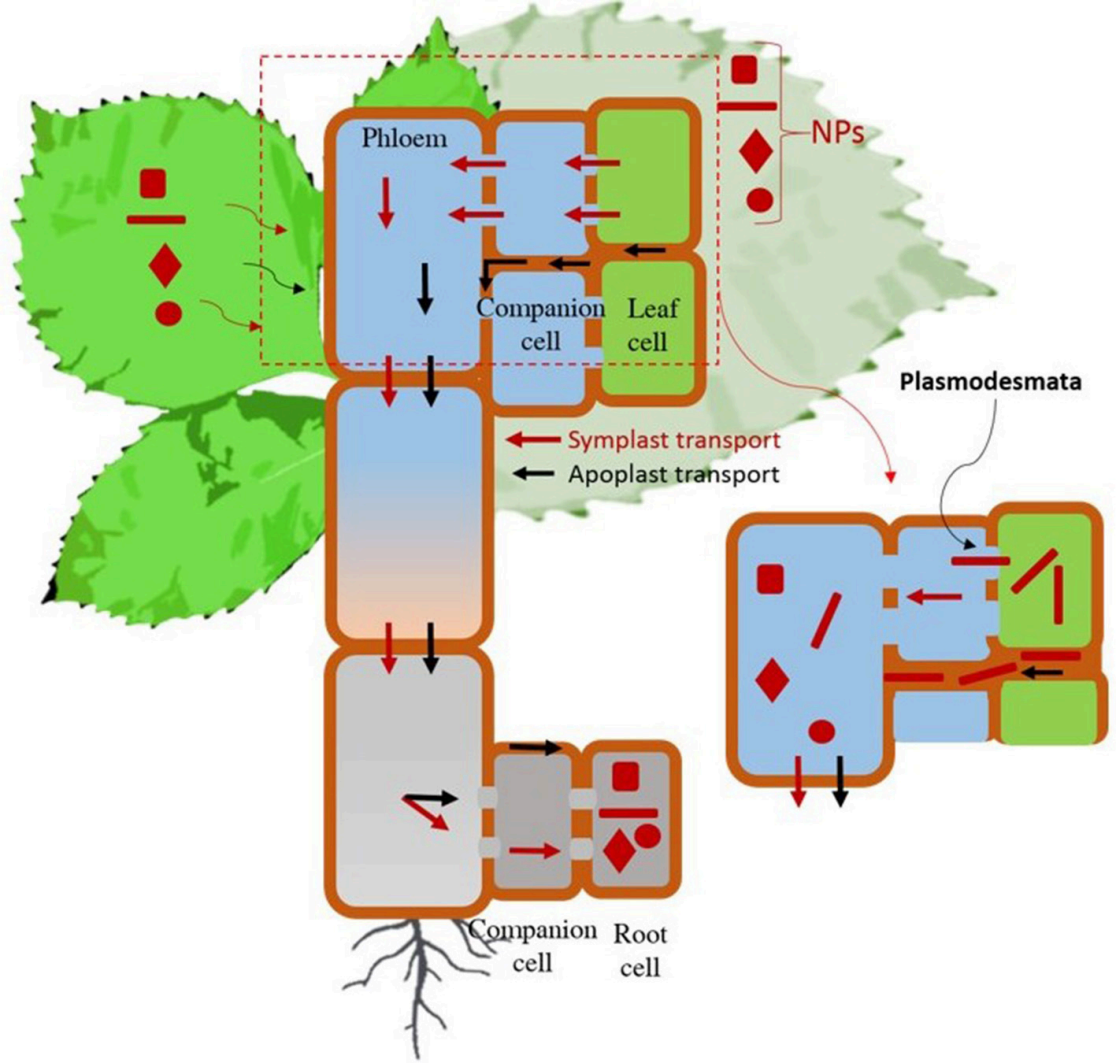
**Mechanistic understanding of nanoparticle transport within plant cells**. Representation describes how nanoparticles transport through Apoplast and Symplast pathway in plants cells along with pressure gradient or mass flow of photosynthate product. Inset represent the favorable transport of gold nanostructure (rod shape) more through Apoplast than Symplastic pathway. NPs, nanoparticles. Color gradient in the phloem represents mass concentration of photosynthate with nanoparticles.

Foliar application increases uptake of nanoparticles by bypassing the cuticle, the primary barrier (Wang et al., 2013), although cuticle repels polar substances (Schwab et al., 2015). The stomatal pore in a typical plant leaf is approximately 100 nm in diameter (Schwab et al., 2015), presents a relatively large gap for the weak cuticle charge to sustain complete anion repulsion (Eichert and Goldbach, 2008). Aaerosol application method surpasses the cuticle barrier by delivering nanoparticles through the stomatal openings (Figure 5B). The subsequent transport of Au NPs from shoot to root is then achieved by plant's vascular systems, portrayed schematically in Figure 6. Cellular transport of nanoparticles carried out by both apoplast (through the wall) and symplast (cell to the cell, mediated by plasmodesmata). Apoplast pathway favors transport of larger particles (~200 nm) but symplast pathway favors smaller (< 50 nm) particles (Schwab et al., 2015). The cellular transport limitation suggests that apoplast pathway is dominant for drop cast methods, whereas, symplast is more common for aerosol mediated delivery of nanoparticles. It also explains the reason for more transport of rod shape particles to roots though drop cast than aerosol approach. The rod-shaped NPs that penetrated or were internalized in the cell have more probability to remain in the plasmodesmata due to the high aspect (inset of Figure 6). Once nanoparticles were internalized further, transport took place through the vascular system of the phloem. The pressure gradient of photosynthate in leaves driven a flow stream of nanoparticles and assist to move in phloem through phloem loading mechanism (Giaquinta, 1983). This pathway of nanoparticle transport through the xylem and phloem has been verified previously (Wang et al., 2012; Raliya et al., 2015). As a result of vascular transport of Au nanoparticles in watermelon plants, they were found to accumulate in the root, stem and leaf to varying degrees, both due to particle morphology as well as the nanoparticle delivery technique. The study opens the door for investigation to use compatible metal nanostructure for targeted delivery of genetic material to plants.

In summary, cellular uptake and accumulation of gold nanoparticles within *C. lanatus* was confirmed by ICP-MS after delivery through their leaf surface. Translocation of nanoparticles from leaf to root showed evidence that nanoparticles travel by the phloem transport mechanism. From the morphology-dependent trends in nanoparticle translocation, it can be concluded that accumulation and transport of nanoparticles depends on nanoparticle shape. Such trends were also influenced by the application method used. Application of nanoparticles by the aerosol method was most effective with structures with low aspect ratio, resulting in greater translocation than application by the drop-cast method. Conversely, the drop-cast method resulted in higher translocation of nanorods, which had a higher aspect ratio than other morphologies tested. This evidence suggests that different application methods may be optimal for delivery of different morphologies of nanoparticles to plants.

## Author contributions

RR, CF did the plant exposure experiment. RR, CF and SC synthesize and characterize the gold nanoparticles. SC, CF, RN and NR contributed in ICP MS analyses. PB help in overall experimental plan and suggestion that driven the study to conclusion. RR, CF wrote the manuscript and others reviewed the draft.

### Conflict of interest statement

The authors declare that the research was conducted in the absence of any commercial or financial relationships that could be construed as a potential conflict of interest.
